# Emerging roles of a chemoattractant receptor GPR15 and ligands in pathophysiology

**DOI:** 10.3389/fimmu.2023.1179456

**Published:** 2023-06-30

**Authors:** Yukari Okamoto, Sojin Shikano

**Affiliations:** Department of Biochemistry and Molecular Genetics, University of Illinois at Chicago, Chicago, IL, United States

**Keywords:** GPR15, C10orf99, GPCR, chemokine, homing, inflammation

## Abstract

Chemokine receptors play a central role in the maintenance of immune homeostasis and development of inflammation by directing leukocyte migration to tissues. GPR15 is a G protein-coupled receptor (GPCR) that was initially known as a co-receptor for human immunodeficiency virus (HIV) and simian immunodeficiency virus (SIV), with structural similarity to other members of the chemoattractant receptor family. Since the discovery of its novel function as a colon-homing receptor of T cells in mice a decade ago, GPR15 has been rapidly gaining attention for its involvement in a variety of inflammatory and immune disorders. The recent identification of its natural ligand C10orf99, a chemokine-like polypeptide strongly expressed in gastrointestinal tissues, has established that GPR15-C10orf99 is a novel signaling axis that controls intestinal homeostasis and inflammation through the migration of immune cells. In addition, it has been demonstrated that C10orf99-independent functions of GPR15 and GPR15-independent activities of C10orf99 also play significant roles in the pathophysiology. Therefore, GPR15 and its ligands are potential therapeutic targets. To provide a basis for the future development of GPR15- or GPR15 ligand-targeted therapeutics, we have summarized the latest advances in the role of GPR15 and its ligands in human diseases as well as the molecular mechanisms that regulate GPR15 expression and functions.

## Introduction

The major task of the intestinal immune system is to tolerate innocuous food antigens and commensal microbes while fighting ingested pathogens. Failure to balance tolerogenic and inflammatory reactions can result in diseases, such as inflammatory bowel disease and gastrointestinal infections. Multiple immune mechanisms, including the balanced activities of regulatory T (Treg) cells and effector T (Teff) cells, contribute to the maintenance of intestinal immune homeostasis. Trafficking of these immune cells to the intestines is tightly controlled by a wide array of chemokines, chemokine receptors, and adhesion molecules expressed by leukocytes, the vascular endothelium, and the epithelium (1). For instance, the expression of CCR5, CCR6, CCR9, and CCR10 together with α4β7 integrin has been shown to target lymphocytes in the small intestine and colon (2–5). One of the recent advances in this field is the identification of G-protein coupled receptor 15 (GPR15) as a new chemoattractant receptor that mediates the homing of T cells to the colon in response to the natural ligand C10orf99 produced in the colon (6–8). A growing number of studies have suggested important roles of GPR15 in immune homeostasis and pathology of gastrointestinal (GI) tissues (6, 9–11). Similarly important is the discovery of GPR15-independent activity of C10orf99, as well as additional GPR15 ligands, that appear to be involved in the pathology of a broader range of tissues. Collectively, these findings indicate that GPR15 and its ligands are promising new targets for intervention. However, currently there is no approved drug that can specifically modulate GPR15 or GPR15 ligand activities. This review aimed to provide a basis for the development of GPR15- or GPR15 ligand-targeted interventions by summarizing recent research advances in the roles of GPR15 and its ligands in human pathophysiology, as well as the regulatory mechanisms of GPR15 expression and functions.

## GPR15

GPR15 is a member of the Class A GPCR family that was cloned in 1996 (12) and identified in 1997 as a co-receptor for SIV, macrophage-tropic, non-syncytium-inducing HIV type 1 (M-tropic HIV-1), and HIV-2 (13, 14). Although this receptor was found to mediate T cell trafficking to the colon (6) and later “deorphanized” when a chemokine-like protein C10orf99 was identified as a functional endogenous ligand (7, 8), GPR15 is an “orphan” in terms of relatively low sequence similarity to its paralogues; it resembles a probable orphan receptor GPR25 with highest similarity of 36%, which is marginally higher than that to angiotensin II receptors, apelin receptor, and other chemokine receptor members (https://www.ensembl.org). GPR15 is also unique in that it lacks cysteines in the NH2-terminal region and the third extracellular loop, which are thought to form a disulfide bond and are required for optimal ligand binding and/or receptor activation in many GPCRs (15). Nevertheless, the NH2-terminal region of GPR15 carries several Tyr and acidic residues, a feature shared by multiple chemokine receptors (16). Sulfation of Tyr residues is known to promote the receptor binding of HIV/SIV (16, 17) as well as chemokine ligands (18–20), and the sulfated Tyr residues in the GPR15 NH2 terminus were recently shown to be required for optimal binding to C10orf99 (21). In humans, *Gpr15* mRNA is highly expressed in the colon and lymphoid tissues, including peripheral blood lymphocytes and the spleen (13), while different studies have reported GPR15 protein expression in the colonic and small bowel mucosa, lymphoid cells, testis, liver, prostate, vascular endothelium, and skin (7, 22, 23). In peripheral blood, GPR15 is expressed in T cells (primarily CD4^+^) and at lower levels in B cells, monocytes, and neutrophils (11, 24, 25). It is of note that GPR15 expression was not induced by retinoic acid (6), that is known to regulate lymphocyte migration to the small intestine by enhancing the expression of CCR9 and integrin α4β7 (26, 27). This may contribute to the colon-specificity of GPR15^+^ cell homing, combined with the abundant expression of C10orf99 in the colon compared with the small intestine (7, 8).

## Role of GPR15 in disease pathology

### GPR15 expression in mouse T cell subsets and colitis

A novel function of GPR15 as a colon-homing receptor was discovered in 2013 by Kim et al. using *Gpr15*-deficient mice (6). Knock-in mice with *Gpr15* gene replaced with the GFP sequence showed preferential expression of GFP in Foxp3^+^ Tregs in the large intestine lamina propria (LILP); approximately 60–70% of LILP CD4^+^Foxp3^+^ cells expressed GPR15, compared to 7–20% of CD4^+^Foxp3^−^ cells. The results of a cell transfer assay confirmed the efficient homing of GPR15^+^ cells to LILP in an α4β7-dependent manner. The *Gpr15* gene knockout (KO) reduced Treg numbers in LILP and exacerbated colitis induced by *Citrobacter rodentium* infection. In addition, in a non-infectious colitis model in *Rag2* ^-/-^ mice, where CD40 stimulation induced innate immune cell-mediated colitis (28–30), adoptive transfer of Tregs from wild-type mice reduced colitis severity and tissue damage, but Tregs from *Gpr15* KO mice failed to do so (6). These observations suggest that GPR15 is required to dampen the immune response in the large intestine by directing homing of Tregs in mice.

However, a subsequent study by Nguyen et al. using *Gpr15* KO mice demonstrated that GPR15 is also important for the colon migration of pathogenic Teff cells that cause inflammation (11). In a T cell-mediated colitis model in which adoptive transfer of CD45RB^hi^ T cells (naïve CD4 T cells depleted of Tregs) to immunodeficient recipient mice resulted in the generation and intestinal trafficking of Teff cells to cause colitis (31–33), *Rag2* ^−/−^ mice that received naïve *Gpr15-*KO T cells were protected from developing colitis. In addition, GPR15 was found to be induced in *in vitro*–generated mouse Th17 effector cells under conventional polarizing conditions (11). Moreover, a more recent study by Xiong et al. clearly showed substantial GPR15 expression in all Th1, Th2, and Th17 subsets isolated from mouse LILP, although at a lower frequency compared with that of Treg cells (34). Hence, GPR15 is capable of directing the colon homing of both Treg and Teff CD4^+^ T cells in mice, and the impact of this receptor in colitis pathology will depend on the experimental settings regarding the relative requirement of Treg and Teff subsets for the development of colitis.

### GPR15 expression in human T cell subsets and colitis

How are these mouse studies translatable to humans? The original study by Kim et al., who discovered the colon homing function of GPR15 in mouse Tregs, observed an increased amount of *Gpr15* mRNA in the human CD25^−^CD4^+^ T cell population than in Treg-enriched CD25^+^CD4^+^ T cells from colon tissues of patients with colorectal cancer (6), implying a difference between mice and humans. The following study by Nguyen et al. found that in human colon tissues, GPR15 expression was highly enriched in the IL-5^+^ or IL-13^+^ Th2 subset, particularly in patients with ulcerative colitis (UC), and there was little or no GPR15 expression in Treg cells (11). *In vitro* polarization of human peripheral blood mononuclear cells (PBMCs) and mouse spleen cells by cytokines also revealed disparate GPR15 expression patterns in T cell subsets between the two species; GPR15 was expressed primarily in Th2 cells in humans, whereas GPR15 was expressed in Treg and Th17 subsets in mice (11). Further analyses of *Gpr15* gene and master T cell transcription factors for Th2 (GATA3) and Treg cells (Foxp3) led to the conclusion that the following mechanism underlies preferential expression of GPR15 in Th2 cells in humans: (i) GATA3 promotes *Gpr15* gene expression in human Th2 cells by binding to the 3′ enhancer of *Gpr15*, while (ii) Foxp3 binding to the enhancer suppresses *Gpr15* expression in human Treg cells, and (iii) this GATA3 binding does not occur in mouse Th2 cells and Foxp3 binding is much weaker in mouse Treg cells because of the sequence difference in the *Gpr15* enhancer. These authors concluded that GPR15 is preferentially expressed in Teff cells rather than in Tregs in humans and this is reflected in the inflamed colon of patients with UC.

In another study, Fischer et al. utilized a humanized mouse model to comparatively determine the role of GPR15 and α4β7 integrin, in which human peripheral blood T cells from patients with UC were transferred into mice and examined for migration to the colon in dextran sulfate sodium (DSS)-induced colitis (35). Expression of both GPR15 and α4β7 integrin was elevated in Tregs but not in Teff cells in the colon of patients with UC compared with those in healthy controls and patients with CD. Pre-treatment of these cells with siRNA for GPR15 only affected Teff cell homing but did not affect Treg homing, whereas treatment with an α4β7 antibody (vedolizumab) suppressed both Teff and Treg homing, suggesting that GPR15 is important for Teff homing but not Treg homing in humans (35).

Despite all these findings suggesting species differences, a simplified interpretation such as “GPR15 is expressed by Teff cells in humans, while it is expressed by Treg cells in mice,” requires caution. Indeed, multiple studies from different groups, including later studies from the same group of Nguyen et al., have shown that GPR15 is expressed by human Tregs in the peripheral blood at similar or even higher levels than that in Teff cells (6, 7, 35–37), which is not consistent with the generalized model that GPR15 expression is promoted in Th2 cells and suppressed in Tregs in humans. Furthermore, Adamczyk et al. reported that GPR15 was expressed at an even lower frequency in Teff cells than in Treg cells in colon tissues of either healthy controls or patients with UC (36). Interestingly, they observed significantly increased expression of GPR15 in both Treg and Teff cells from the uninflamed region, but not from the inflamed region, in patients with UC compared with that in tissues from healthy controls. This is also consistent with a more recent study by Xiong et al. that observed substantial expression of GPR15 (nearly 40% on average) in Tregs from uninflamed regions of UC patient colons and a positive correlation between GPR15 and Foxp3 expression in human colonic Tregs (34). These findings are in contrast to the predominant GPR15 expression by Th2 cells observed in UC colon by Nguyen et al. (11). The reason for this discrepancy is not clear, and further human studies are necessary; however, the differential expression of GPR15 in Tregs in the inflamed and uninflamed regions of the UC colon could provide some insights. The fact that GPR15 expression was not increased in Tregs from the inflamed region of the UC colon implies that GPR15 is not an exclusive master regulator for the migration of Tregs into the inflamed colon. On the other hand, the increased GPR15 expression on Tregs in the non-inflamed region also raises the possibility that GPR15 drives Tregs to the colon of patients with UC, but its expression is downregulated in the inflamed environment. For instance, increased local production of the GPR15 ligand might enhance receptor internalization and subsequent degradation. In addition, since dysbiosis in UC is characterized by reduced levels of bacterial metabolites, including short chain fatty acids (SCFAs) (38) and GPR15 expression was found to be upregulated by SCFAs (36, 39, 40), the diminished local production of SCFAs could result in the reduced expression of GPR15 on Tregs in the inflamed colons of patients with UC. Interestingly, the aforementioned study by Adamczyk et al. (36) found that the majority of Teffs and Tregs in the peripheral blood did not co-express GPR15 and α4β7 integrin, and this lack of co-expression was also detectable in colonic biopsies of healthy individuals and patients with UC. This indicates the phenotypic heterogeneity of T cells, especially Tregs (41, 42) and highlights the need for careful analysis of the expression of multiple molecular factors potentially involved in the colonic migration of T cells.

### Colorectal cancer

In addition to colitis, GPR15 has also been implicated in the pathogenesis of CRC. Tregs infiltrating the tumor sites create an immunosuppressive tumor microenvironment that prevents the development of effective anti-tumor immune responses (42, 43). Adamczyk et al. compared gene expression in Treg cells from tumors and healthy colonic tissues of colitis-associated CRC mice induced by azoxymethane (AOM)/DSS treatment (10). They identified a specific set of genes that are preferentially expressed in tumor-associated Tregs, including GPR15. Similar to mice, the frequency of GPR15^+^ Tregs, but not that of GPR15^+^ Teffs, in the tumor sites of patients with CRC was significantly higher than that in non-tumor sites, suggesting a distinct role for GPR15 in Treg delivery to CRC sites in humans (10). Importantly, genetic deletion of *Gpr15* in mice significantly decreased the infiltration of tumor-associated Tregs, reduced the Treg/CD8^+^ T cell ratio, and diminished tumor development (10), suggesting that GPR15 is responsible for directing the colon migration of Treg cells that support the growth of CRC. Thus, GPR15 represents a promising novel target for modifying T cell-mediated anti-tumor immunity in CRC.

### Eosinophilic esophagitis

EoE is an allergic disease characterized by chronic esophageal inflammation with prominent recruitment of eosinophils (44). Inflammation in EoE critically involves Th2 cells that produce cytokines such as IL-5 and IL-13, which promote eosinophil recruitment and activation and exacerbate epithelial barrier dysfunction, respectively (44, 45). In a recent study, Morgan et al. (9) conducted single-cell RNA analysis of T cells in tissues from patients with EoE. They found that *Gpr15* expression was increased in highly polarized pathogenic effector Th2 (peTh2) clonotypes detected in both esophageal tissue and peripheral blood of patients with EoE, and *Gpr15* was the most significantly upregulated transcript in these cells in the esophagus compared with peripheral blood. While the genes encoding integrin α4β7 were broadly expressed by T cells in both the esophagus and duodenum, GPR15 was only expressed in esophageal T cells, and CCR9, a chemokine receptor known for gut homing (46), was only expressed in duodenal T cells. In addition, the authors detected the expression of C10orf99 and CCR9 ligand CCL25 only in the esophageal epithelium and duodenal epithelium, respectively (9). This is consistent with the fact that the esophagus is one of the tissues that most strongly expresses C10orf99 (https://gtexportal.org). These findings collectively support the model that GPR15 expression promotes esophageal homing of peTh2 cells and exacerbates inflammation during EoE, and in addition, GPR15 may serve as a marker for esophagus-migrating peTh2 cells in the peripheral blood of patients.

### Rheumatoid arthritis

An earlier study on RA reported that GPR15 is expressed by macrophages in synovial tissue and monocytes and neutrophils in peripheral blood, and its expression is upregulated in patients with RA compared to non-RA controls (24). A more recent study examined GPR15 expression on T cells from patients with RA and found that the frequency of CD4^+^/CD8^+^ GPR15^+^ T lymphocytes was higher in patients with RA than in healthy subjects (47). In addition, the frequency of CD4^+^/CD8^+^ GPR15^+^ T lymphocytes was higher in the synovial fluid of patients with RA than in that of patients with osteoarthritis. Immunostaining results of synovial tissue sections demonstrated that GPR15 and GPR15L are present in the synovial tissues of patients with RA (47). These findings implicate the GPR15-GPR15L axis in RA pathogenesis, which involves both innate and adaptive immune cells.

### Skin inflammation

The mouse skin epithelium contains a specialized population of γδ T cell receptor (TCR)^+^ cells called dendritic epidermal T cells (DETCs), which exclusively express the monoclonal Vγ3Vδ1 TCR and are implicated in protection of skin homeostasis, host defense, and wound healing (48). DETCs mature in the fetal thymus and migrate to the skin during late embryogenesis, after which they are maintained through self-renewal. An earlier study by Lahl et al. found that GPR15 is highly expressed on fetal thymic DETC precursors and that *Gpr15* KO substantially reduces the frequency of epidermal DETCs in neonatal (Day 1) mice compared with *Gpr15* ^-/+^ heterozygote neonates (23). This suggested that GPR15 is essential for migration of embryonic DETC to the skin, which is also consistent with the high expression of *C10orf99* in keratinocytes of embryonic (day 16) and neonatal skin (7). In a more recent study, Sezin et al. conducted a profiling of T cell populations in the skin of adult (8-16 weeks) *Gpr15* KO mice and found that the DETCs were reduced by approximately 60% compared to wild-type littermates (49). In addition, the niche of DETCs in the epidermis was populated by αβ TCR^+^ cells; approximately 40% of all CD3^+^ cells in *Gpr15* KO mice were αβ TCR^+^ compared to only 10% in wild-type mice (49). Furthermore, these changes were also associated with shifts in the composition of skin microbiome in *Gpr*15 KO mice (49). These studies collectively highlighted a pivotal role of GPR15 in the skin homing of DETCs in mice, which appears to impact the composition of T cell populations and microbiome even in the adulthood.

On the other hand, the role of GPR15 in the skin disease settings remain somewhat elusive. Deficiency in *Gpr15* did not alter the course of disease neither in the imiquimod-induced psoriasiform dermatitis nor in the IL-23-induced dermatitis model, despite the increased expression of C10orf99 in the inflamed skin (50). However, in the antibody transfer mouse model of bullous pemphigoid-like epidermolysis bullosa acquisita (BP-like EBA), an autoimmune subepithelial and mucocutaneous blistering disease, the *Gpr15* KO was found to markedly aggravate the skin pathology (51). Importantly, this was associated with an increased accumulation of γδ TCR^+^ cells in the dermis (51), suggesting a possibility that GPR15 may counteract antibody-mediated skin inflammation through direct and/or indirect mechanisms that limit the recruitment of γδ TCR^+^ cells into the dermis.

## GPR15 ligands and their roles in pathophysiology

### C10orf99 in GPR15 signaling

**Figure 1:**
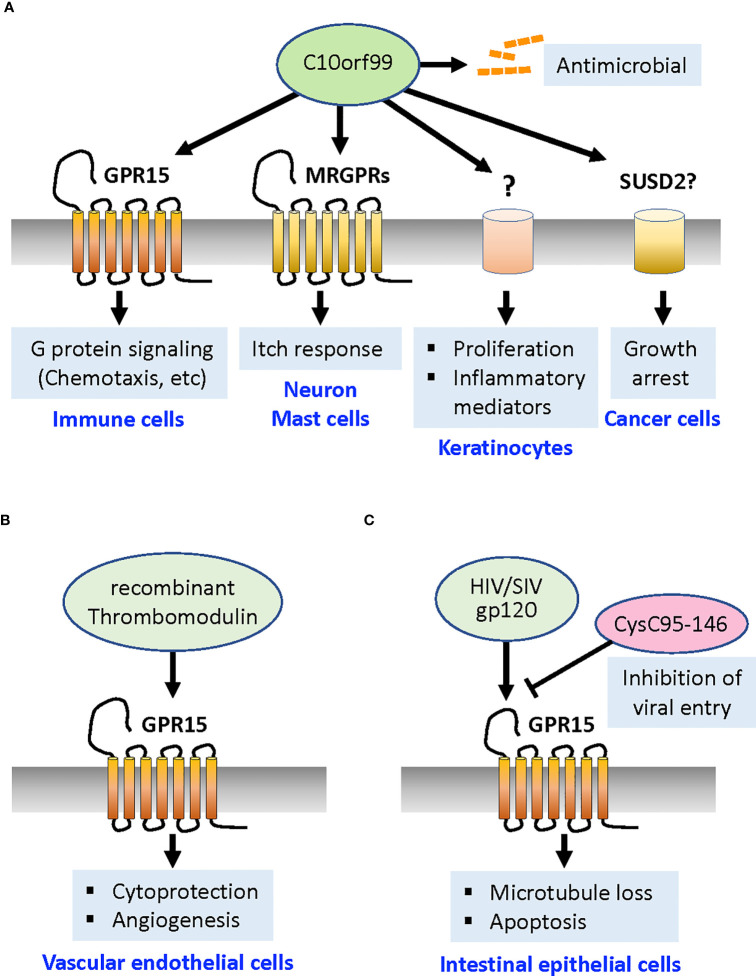
GPR15 ligands and their functions. **(A)** A natural GPR15 ligand C10orf99 activates the Gαi-mediated signaling pathways that induce chemotaxis of GPR15^+^ T cells and other immune cells toward this ligand. C10orf99 also functions independently of GPR15 to exert antimicrobial activities. In the psoriatic skin, C10orf99 evokes itch response by activation of MRGPRs on sensory neurons and mast cells, and promote proliferation and production of inflammatory mediators of keratinocytes through unknown receptor/mechanism. C10orf99 also induces growth arrest of cancer cells by activating SUSD2 receptor (although controversial). **(B)** Recombinant thrombomodulin acts on GPR15 expressed by vascular endothelial cells via its fifth region of EGF-like domain to confer cytoprotection from apoptosis signal and promote angiogenesis. **(C)** HIV protein gp120 induces loss of microtubule and apoptosis in the intestinal epithelial cells. The C-terminal fragment of cystatin C (CysC95-146) competes for GPR15 and inhibits the entry of HIV and SIV. *MRGPRs*, Mas-related G protein-coupled receptors; *SUSD2*, Sushi Domain Containing 2 receptor; *HIV*, human immunodeficiency virus; SIV, simian immunodeficiency virus.

C10orf99 was reported as a natural GPR15 agonist in 2017 (7, 8). Mature human C10orf99 is a short, 57-amino acid basic protein (pI = 11.28) with two pairs of Cys residues that form intramolecular disulfide bridges, implying that this protein is related to a CC chemokine. The tissues expressing C10orf99 include the digestive tract (particularly the colon and esophagus), skin, tonsils, cervix, and bladder. In the mouse colon, C10orf99 expression did not appear to be regulated by colonic inflammation or the presence of commensal bacteria (7). C10orf99 has several unique features that differ from those of the canonical chemokines. Secondary structures typically found in chemokine family proteins, such as loops, β-strands, and helices, were not identified in C10orf99 by structure prediction programs (8). In contrast to most chemokines that require their N-termini for receptor binding and activation (52), N-terminal deletion of C10orf99 by up to 10 amino acids showed no marked change in GPR15-dependent calcium signaling (8). Instead, the hydrophobic C-terminal region of C10orf99, which is highly conserved among species, is critically required for receptor binding and signaling (8, 21, 53). In addition, unlike canonical chemokines and chemokine receptors, C10orf99 and GPR15 show highly specific interactions; C10orf99 does not cross-activate any of the known 22 chemokine receptors, and GPR15 does not respond to any of the 27 known chemokines (8). Functionally, C10orf99 interaction with GPR15 leads to inhibition of cAMP production, which can be reversed by pertussis toxin, indicating that GPR15 is a Gαi/o-coupled receptor, similar to most chemokine receptors. C10orf99 activates extracellular signal-regulated kinase (ERK)1/2, induces calcium release, promotes β-arrestin recruitment and receptor endocytosis, and induces chemotaxis in GPR15-expressing immune cells (7, 8, 21, 53, 54) (**Figure 1A**).

### C10orf99 in psoriasis

C10orf99 was implicated in immune regulation prior to the discovery of its GPR15 agonistic activity. Its first implication in human diseases came from global analyses of psoriasis-associated genes in 2009 which reported significant upregulation of *C10orf99* in psoriatic skin (55, 56). A large-scale gene knockout study in 2010 showed that disruption of *C10orf99* gene leads to an increased ratio of CD4^+^/CD8^+^ cells and a decreased serum IgM level in mice (57). Multiple studies have demonstrated the regulated expression of *C10orf99* in the epidermis of the skin and its association with psoriasis. Consistent with the epidermal migration of fetal thymic DETCs that express GPR15 (23), *C10orf99* mRNA is highly expressed in the fetal and neonatal epidermis of mice, but is low or nearly absent in the uninflamed adult epidermis both in mouse and human (7, 51). However, *C10orf99* expression was highly upregulated in wounded skin (7), antibody-mediated model of BP-like EBA (51), and imiquimod-induced models of psoriasis in mice (8, 51, 58), and in patients with psoriasis (8, 58–60) or atopic dermatitis (59, 61). *C10orf99* has also been indicated as a reliable marker gene for the classification of psoriasis (60).

A major question is whether C10orf99 plays a determining role in disease progression or whether it merely indicates loss of epithelial integrity. It appears that C10orf99 has GPR15-independent functions. Yang et al. reported that C10orf99 acts as an antimicrobial peptide that exhibits broad-spectrum antimicrobial activity (62) (**Figure 1A**), as has been reported for some chemokines (63). In addition, local sustained delivery of nanoparticle-encapsulated C10orf99 peptide promoted granulation tissue formation and wound healing in a full-thickness dermal defect rat model (64). Similarly, overexpression of *C10orf99* gene in transgenic mouse was reported to reduce skin inflammation and remodeling after an imiquimod challenge in a published patent application (65). These findings implicate a protective role for C10orf99 in the inflammatory skin.

However, a later study using human keratinocyte HaCaT cells under inflammatory conditions showed that *C10orf99* knockdown decreased cell proliferation, whereas overexpression of C10orf99 promoted their proliferation (58) (**Figure 1A**). A more recent study also showed that *C10orf99* transfection into normal human epidermal keratinocytes induced the expression of inflammatory mediators and reduced the expression of barrier-related genes such as filaggrin and loricrin (59). The addition of synthetic C10orf99 peptide reduced the expression levels of barrier-related genes in human keratinocyte 3D cultures, suggesting that C10orf99 binding induces specific signaling in keratinocytes (59). Furthermore, local depletion of *C10orf99* by lentiviral shRNA vectors or systemic knockout of *C10orf99* in mice effectively ameliorated imiquimod-induced psoriatic dermatitis, supporting the proinflammatory role of C10orf99 (58, 59). Collectively, these results indicate that C10orf99 is a primary inducible regulator that reduces barrier formation and induces the inflammatory response of keratinocytes under psoriatic conditions (**Figure 1A**). As already mentioned, deficiency in the *Gpr15* gene did not alter the course of disease in imiquimod-induced psoriasiform dermatitis or the IL-23-induced dermatitis model, suggesting that C10orf99 modulates psoriasiform dermatitis via GPR15-independent pathways (50).

The notion of GPR15-independent activity of C10orf99 is further supported by a recent study by Tseng and Hoon (61) who discovered that C10orf99 can act as an endogenous pruritogen during inflammation that activates Mas-related G protein-coupled receptors (MRGPRs). These authors found that C10orf99 selectively stimulates mouse dorsal root ganglion neurons that express Mrgpra3 and evokes intense itch responses and vasodilation. C10orf99 also caused mast cell degranulation through the stimulation of MRGPRX2 and Mrgprb2, and genetic disruption of *C10orf99* expression attenuated scratch responses in an imiquimod-induced psoriasis model (61). Together, these studies suggest that elevated expression of C10orf99 during psoriasis can aggravate the disease by promoting the proliferation and inflammatory response of keratinocytes, reducing barrier formation, and inducing itch responses and vasodilation by acting on neurons and mast cells (**Figure 1A**).

### C10orf99 in cancer cell growth

The above-mentioned studies indicate that C10orf99 has multiple receptors and cellular substrates that are involved in physiologically different reactions. An earlier study by Pang et al. reported another function of C10orf99: the growth inhibition of cancer cells (66). C10orf99 was found to interact with the transmembrane protein Sushi Domain Containing 2 (SUSD2), hence termed a colon-derived SUSD2 binding factor (CSBF) (66). The authors showed that the C-terminally IgG-Fc-tagged recombinant C10orf99/CSFB protein binds to SUSD2 expressed in CRC cell lines and inhibits cell growth through G1 cell cycle arrest (66) (**Figure 1A**). However, this model requires further investigation because the inhibitory effect of C10orf99 on CRC cells could not be reproduced in a follow-up study by a different group (62) who used the untagged C10orf99 protein and observed its cytotoxic effect only on a specific B-cell lymphoma line.

### Thrombomodulin as a ligand to GPR15 in vascular endothelial cells

A novel function of GPR15 in ECs, mediated by a ligand that is completely distinct from C10orf99 in structure, was demonstrated in 2017 (67) (see more comprehensive review (68)). Pan et al. found that the recombinant soluble protein TME5, which encodes the fifth region of the epidermal growth factor (EGF)-like domain of TM, binds to the GPR15 expressed by human vascular ECs *in vitro* (**Figure 1B**). TM is a type I transmembrane protein constitutively expressed by ECs and acts as an anticoagulant by binding to thrombin and activated protein C (69). The blood level of soluble TM is known to be elevated under various pathological conditions involving endothelial damage, such as sepsis, COVID-19 infection, progressive systemic sclerosis, and diabetes (70–73). Recombinant human soluble TM (rTM, ART-123), consisting of the extracellular domain of TM, has been used to treat disseminated intravascular coagulation (DIC) (74, 75). Pan et al. demonstrated that recombinant TME5 rescued growth inhibition and apoptosis caused by the calcineurin inhibitor FK506 in vascular ECs isolated from wild-type but not from FK506-treated *Gpr15* KO mice (67). This cytoprotective effect was mediated by the activation of ERK1/2 and increased level of anti-apoptotic proteins (67). In addition, TME5 enhanced the migratory activity of ECs and increased their production of nitrogen oxide. Moreover, *in vivo* Matrigel plug angiogenesis assay revealed that TME5 stimulates angiogenesis in wild-type mice but not in *Gpr15* KO mice (67) (**Figure 1B**). TME5 also ameliorates inflammation in a murine sepsis model in a GPR15-dependent manner through suppression of NF-kB activity and release of pro-inflammatory cytokines in macrophages (76). GPR15 in T cells also appears to mediate TME5-induced anti-inflammatory effects in a murine model of acute graft-versus-host disease (GVHD) caused by allogeneic hematopoietic stem cell transplantation (77).

Interestingly, in a more recent study testing the potential anti-tumor effect of rTM, it was suggested that GPR15 mediates rTM-induced growth inhibition of pancreatic ductal adenocarcinoma (PDAC) (78). The anti-tumor effect of rTM was only observed in PDACs with high GPR15 expression, and rTM suppressed NF-kB and ERK1/2 activation in a GPR15-dependent manner. This inhibition of ERK1/2 activity by rTM is not consistent with its cytoprotective effect in ECs that involves activation of ERK1/2 (67), suggesting that the consequence of rTM-GPR15 interaction is dependent on the cell context. Further investigations in different cell types are necessary to delineate TM-induced GPR15 signaling pathways.

### HIV gp120 in enteropathy

Chemokine receptors are expressed in numerous non-hematopoietic cells and play important roles beyond chemotaxis of leukocytes, such as development, angiogenesis, and apoptosis (79–81). GPR15 is abundantly expressed on the basolateral surface of the intestinal epithelium, unlike CXCR4 and CCR5, which are present mainly at or near the luminal surface (22). It was previously reported that HIV-1 surface protein gp120 induces calcium signaling, microtubule loss, and physiological changes, including increased paracellular permeability in the intestinal cell line; these changes resemble HIV enteropathy (82, 83). Fantini et al. found that these gp120-induced effects were inhibited by anti-GPR15 neutralizing antibody or selective G protein inhibitor pertussis toxin (22, 84). They also found that GPR15 mediates viral strain-specific gp120-induced calcium signaling at low, physiologically reasonable gp120 concentrations, which are up to 10,000-fold lower gp120 concentrations than those of the principal HIV co-receptors. These findings suggest that gp120 is involved in HIV enteropathy via its interaction with the GPR15. GPR15 has also been implicated in the apoptosis of intestinal epithelial cells in an SIV infection model (85) (**Figure 1C**).

## Regulation of GPR15 expression

**Figure 2:**
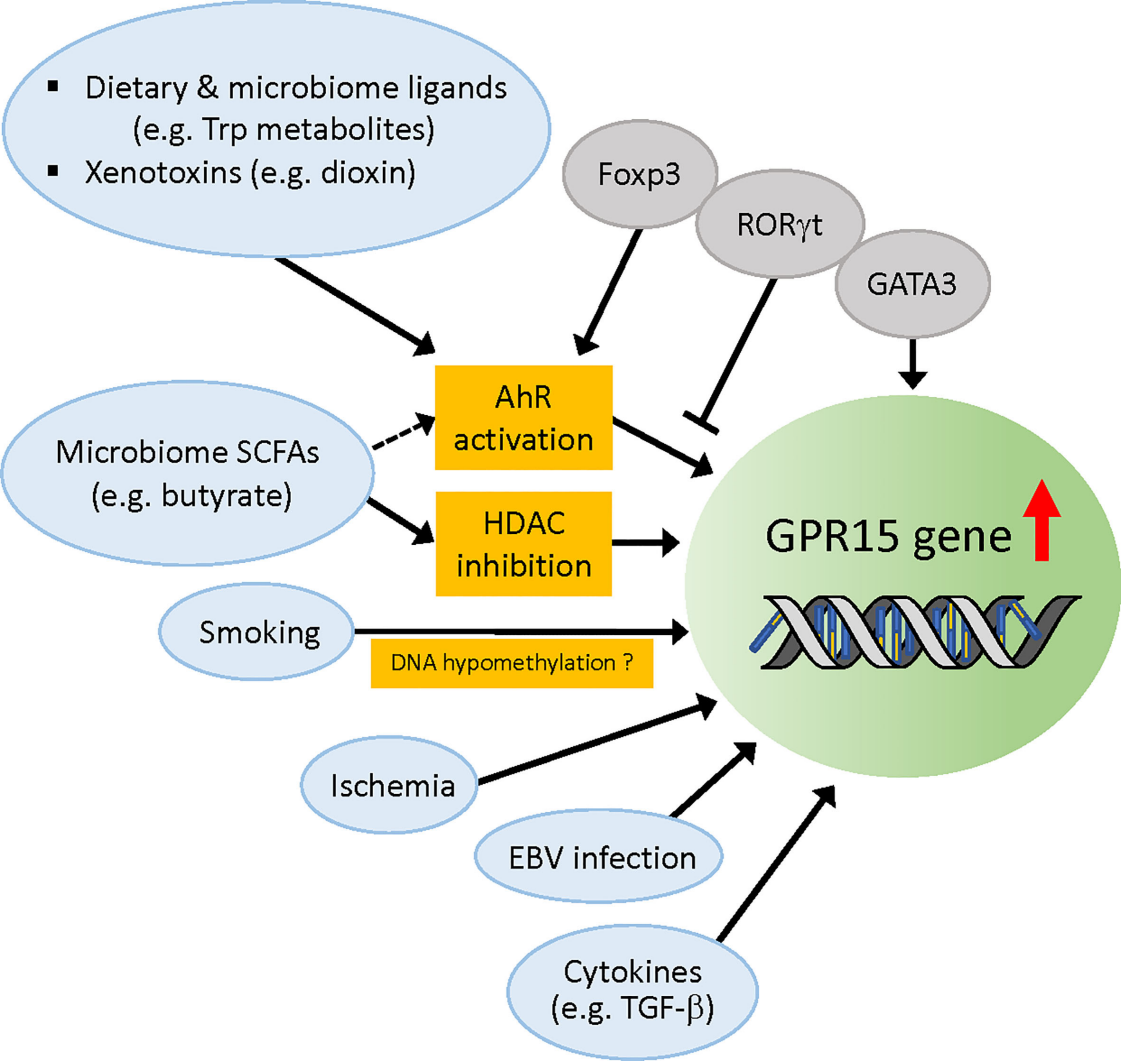
Regulation of *Gpr15* gene expression. Molecular factors that have been reported to upregulate *Gpr15* expression are depicted. Dietary and microbiome ligands such as tryptophan metabolites activate AhR that directly acts on *Gpr15* gene. Foxp3 cooperates with AhR (although contradictory data exist) while RORγt competes with AhR, and GATA3 enhances *Gpr15* expression independently of AhR. Foxp3 and GATA3 are reported to differentially bind *Gpr15* enhancer and regulate *Gpr15* expression in the T cells of human and mouse (not depicted in this figure). SCFAs from microbiota such as butyrate upregulate Gpr15 expression by inhibition of HDAC but might also by activation of AhR. Smoking habit is strongly associated with DNA hypomethylation of *Gpr15* and increased mRNA expression of *Gpr15* in blood leukocytes, but the evidence for the direct effect of cigarette smoke extract on *Gpr15* DNA methylation is lacking. Instead, the increased frequency of peripheral CD3^+^GPR15^+^ T cell population appears to account for the apparent hypomethylation of *Gpr15* DNA in blood samples from smokers. Infarction-related ischemia in mice and ischemic condition in cell culture induces *Gpr15* expression in cardiomyocytes. EBV infection in B cells enhances *Gpr15* expression by an unidentified mechanism. TGF-β induces GPR15 expression *in vivo*. *AhR*, aryl hydrocarbon receptor; *SCFAs*, short chain fatty acids; HDAC, histone deacetylase; *EBV*, Epstein-Barr virus.

The molecular mechanisms that have been reported to regulate *Gpr15* gene expression are depicted in **Figure 2**.

### Regulation of GPR15 expression by aryl hydrocarbon receptor

The AhR is a ligand-activated transcription factor that has been studied for many decades as a sensor for environmental contaminants, such as dioxins. Research in the past 15 years has led to the emergence of AhR as a key physiological regulator of immune responses, affecting both innate and adaptive systems by sensing a variety of endogenous, dietary, microbial (e.g., tryptophan metabolites), and environmental ligands (86). AhR is known to regulate the differentiation, homing, and immunosuppressive functions of Tregs (87, 88) and numerous studies have shown that AhR ligand activation can reduce inflammation and ameliorate disease (89–92). AhR is also downregulated in the intestinal tissue of patients with IBD (93). Using genetic mouse models, Zhou et al. found in 2017 that GPR15 expression is significantly reduced in AhR-deficient colonic Treg cells (94). A more recent study by the same group (34) demonstrated that the AhR-dependency of GPR15 expression was not confined to Tregs, but was observed for all murine CD4^+^ T cell subsets in the colon. In addition, when wild-type mice were fed with an AhR-ligand-deficient diet, the addition of the dietary AhR ligand indole-3-carbinol (I3C) (95) significantly enhanced GPR15 expression in intestinal CD4^+^ T cell subsets, while this effect was abrogated in Tregs (but not in other CD4^+^ subsets) in engineered mice in which *Ahr* was specifically ablated in Tregs (34). Moreover, in a short-term homing assay, in which *in vitro* differentiated Tregs (iTregs) were transferred to *Rag1* ^-/-^ mice, the number of *Ahr^-/-^* Tregs migrating to the colon was approximately 3-fold lower than that of *Ahr^+/+^* Tregs. Importantly, forced expression of GPR15 in *Ahr^-/-^* Tregs significantly enhanced their homing to the colon but not to other organs, suggesting that AhR promotes colon-specific homing of Tregs by enhancing GPR15 expression in mice (34). Notably, GPR15 expression in iTregs generated from human PBMCs was enhanced or reduced by the AhR agonist or antagonist, respectively, indicating that AhR regulates GPR15 in humans as well (34). This is supported by another study by McAleer et al. (96) who found regulation of GPR15 expression by an AhR agonist and antagonist in human CD4^+^ T cells. In a separate recent study, Swaminathan et al. reported the regulation of GPR15 expression by AhR in activated human PBMCs, sorted effector/memory CD4^+^ T cells, and *in vitro* polarized human Th2 and Treg cells (37). Collectively, these studies have uncovered a novel role of AhR in controlling colon homing of CD4^+^ T cells by positively regulating GPR15 expression in mice and humans. It is interesting to note that, retrospectively, there was already an indication of AhR regulation of GPR15 in 2007 when *Gpr15* was identified as a novel dioxin-inducible gene (97).

A recent study demonstrated that the AhR intrinsically promotes differentiation and function of resident memory CD8^+^ T cells, including those in the intestinal epithelium (98). Nevertheless, genetic deletion or activation of AhR did not affect GPR15 expression in CD8^+^ T cells (34), suggesting an AhR-independent mechanism for GPR15 expression in CD8^+^ T cells. The crucial role of AhR in regulating GPR15 expression in CD4^+^ but not in CD8^+^ T cells suggests a potential therapeutic target in intestinal disorders, e.g., colorectal cancer where disruption of *Gpr15* gene reduced infiltration of CD4^+^ Tregs but not CD8^+^ T cells into tumor sites and inhibited tumor growth (10).

### Direct activation of *Gpr15* expression by AhR and regulation by other transcription factors

How does AhR regulate GPR15 expression? Chromatin immunoprecipitation sequencing (ChIP-seq) of AhR using iTregs and Th17 cells identified *Gpr15* as one of the top genome-wide targets of AhR in both cell types (34). In addition, mutations in the DNA-binding region abolished the ability of AhR to promote *Gpr15* expression in iTregs, suggesting that AhR regulates transcription by directly binding to the *Gpr15* locus. Consistent with this notion, both aforementioned studies (34, 37) identified AhR binding sites/xenobiotic response elements (XREs, 5′-GCGTG-3′) within the *Gpr15* 3′ enhancer sequence that are conserved across mammalian species. As expected, the *Gpr15* promoter-driven luciferase reporters attached to these XRE-containing enhancer regions exhibited AhR agonist-dependent activity that was inhibited by an AhR antagonist (34, 37). Further analyses by Xiong et al. (34) revealed that Foxp3 cooperates with AhR, potentially via interactions with AhR at the *Gpr15* locus, to enhance GPR15 expression in Tregs. In contrast, RORγt, which is frequently expressed in gut Tregs and Th17 cells, negatively regulates GPR15 expression, at least in part, by competing with AhR for binding to the *Gpr15* locus (34). In addition, Swaminathan et al. showed that GATA3 and AhR independently and synergistically promoted GPR15 expression (37). Intriguingly, however, these authors also found a significant decrease in GPR15 expression by Foxp3 in the presence of either AhR or GATA3 in the luciferase assay, contrary to the findings of Xiong et al. (34). Although further investigation is necessary to elucidate the crosstalk between AhR and other regulatory factors, the discovery of AhR-mediated regulation of GPR15 expression and colon homing of T cells has significantly advanced our understanding of the roles of GPR15 in the intricate mechanisms of T cell migration to target tissues.

GPR15 expression in T cells can be induced by SCFAs such as butyrate, propionate, and acetate (36, 39, 40), which are the main metabolites produced by the microbiota in colon. These SCFAs synergistically enhance basal and ligand-induced expression of AhR-responsive genes in a gene- and cell context-dependent manner, likely through the inhibition of histone deacetylase (HDAC) (99). Interestingly, a recent study by Marinelli et al. provided evidence suggesting that butyrate acts as an AhR ligand to enhance transcription of AhR ligand-dependent genes, independent of its HDAC inhibitor activity, in intestinal epithelial cell (IEC)-AhR reporter cell lines (100). Although this observation needs to be confirmed in other cell types, the study raises an interesting possibility that SCFA butyrate can upregulate GPR15 expression through activation of AhR in colon-migrating T cells and possibly also in colon epithelial cells.

### Regulation of GPR15 expression by pathophysiological factors

#### Smoking and DNA methylation

A number of genomic analyses of blood cells, mostly methylation analysis, have been performed to identify the molecular targets responsible for smoking-induced reprogramming. Blood T cells and B cells expressing GPR15 have approximately 50% methylation at cg19859270, a CpG site within the single exon of *Gpr15*, while non-GPR15-expressing cells are nearly 100% methylated at this site (101). Many studies have shown that smoking is associated with decreased methylation of cg19859270 (102–108) (also see review (109)) and increased *Gpr15* mRNA expression (105, 107, 108, 110, 111) in blood leukocytes. Smoking was also shown to increase the frequency of peripheral GPR15^+^CD3^+^ T cells (107, 112). The effect of smoking is slowly reversible after cessation (102, 110, 113). These data suggest a potential mechanism by which smoking decreases *Gpr15* DNA methylation, which leads to an increase in *Gpr15* mRNA expression, resulting in increased GPR15-positive T cells in the blood. However, although it is conceivable that hypomethylation of *Gpr15* DNA *per se* contributes to the increased expression of *Gpr15* mRNA, the direct causal effect of smoking on *Gpr15* DNA methylation is questionable. By analyzing GPR15 protein expression in leukocyte subtypes, Bauer et al. (107) found that the increased proportion of CD3^+^GPR15^+^ T cells in the blood of smokers was responsible for the apparent smoking-induced hypomethylation of the *GPR15* gene, since cg19859270 hypomethylation was specifically found in GPR15-expressing cells. In addition, treatment of PBMC cultures with aqueous cigarette smoke extract (CSE) did not induce a higher proportion of this T cell subtype, suggesting that DNA hypomethylation at the cg19859270 site observed in smokers did not arise from the direct effect of tobacco smoking compounds on DNA methylation but rather from the enrichment of a smoking-induced GPR15^+^ T cell population in the peripheral blood (107). This study also indicates that the frequency of GPR15^+^ T cells in the blood can be effectively used as a highly reliable biomarker for tobacco smoking. It remains to be elucidated how smoking leads to an increased population of GPR15^+^ T cells independent of CSE, and whether or how the increased GPR15^+^ T cells in the blood impact immune homeostasis and smoke-related disease pathology.

#### Ischemia

In a recent study by Haase et al. (114), transcriptome analysis showed upregulated *Gpr15* mRNA expression and downregulated *Gpr15* DNA methylation in PBMCs from early onset myocardial infarction (MI) individuals compared to controls. The MI risk prediction analysis indicated that the effect of smoking on MI was fully mediated by *Gpr15* mRNA expression; however, the associations between *Gpr15* mRNA expression and *Gpr15* DNA methylation with MI were found to be independent of smoking status. In addition, cardiac *Gpr15* expression was significantly upregulated in a mouse model of infarction-related ischemia (>6-fold increase at five days after MI) as well as in an ischemic cardiomyocyte culture model (4-fold after 24 h induction of ischemic stress) (114). These data imply that GPR15 might play a role, independent of smoking, in the pathogenesis of acute MI and conditions of ischemia, such as artery narrowing by plaques. Interestingly, *Gpr15* KO mice had reduced survival compared to wild-type mice after MI induction (114), which raises the possibility that MI-induced cardiac GPR15 expression represents a protective response to oxidative stress. Further investigation is warranted to elucidate the pathophysiological role of GPR15 in MI. It will be particularly interesting to determine whether and how GPR15 in cardiomyocytes is activated by known or novel ligands to induce signaling.

#### Epstein–Barr virus

The RNAseq data in the GTEx portal (www.gtexportal.org) showed the strongest expression of *Gpr15* in EBV-transformed lymphocytes (median transcripts per million (TPM) was 252.9, while median TPM of whole blood was 1.0) among diverse human tissues. The mechanism of EBV-induced GPR15 expression is yet to be understood. EBV infects the oropharynx but frequently induces B-cell proliferation, which causes tumors in the gut of immunosuppressed individuals, such as transplant recipients treated with immunosuppressive drugs, namely post-transplantation lymphoproliferative disorders (PTLDs) (115, 116). Delecluse et al. found that EBV infection induces integrin α4β7 expression in tonsillar B cells (117). Since α4β7 is the key for homing of B cells to the gastrointestinal tract (118), this study suggests that the induction of α4β7 is one of the mechanisms through which EBV-infected cells enter the gastrointestinal mucosa-associated lymphoid tissue. It will be interesting to determine whether elevated GPR15 expression in EBV-transformed B cells may contribute to their specific homing to the colon by synergizing with α4β7 to enhance the pathogenesis of PTLDs.

#### Cytokines

GPR15 expression can be induced *in vitro* by a combination of TGF-β1 and either IL-6 or IL-21 preferentially in mouse Tregs (6). However, *Il21r^−/−^Il6^−/−^* mice crossed with *Gpr15^gfp/+^* mice had a similar level of GFP expression as that of control mice, suggesting that TGF-β1 is a key regulator of GPR15 expression *in vivo* (6). An earlier study has shown that both TGF-β1 and TGF-β2 can induce *de novo* Foxp3 expression in CD4^+^CD25^–^ cells (119). This raises a possibility that the effect of TGF-β on GPR15 expression may involve the increase of Foxp3 expression that would promote *Gpr15* transcription through enhancement of the AhR activity (34).

## Regulation of GPR15 by posttranslational modifications

### Regulation of cell surface expression of GPR15 by phosphorylation

**Figure 3:**
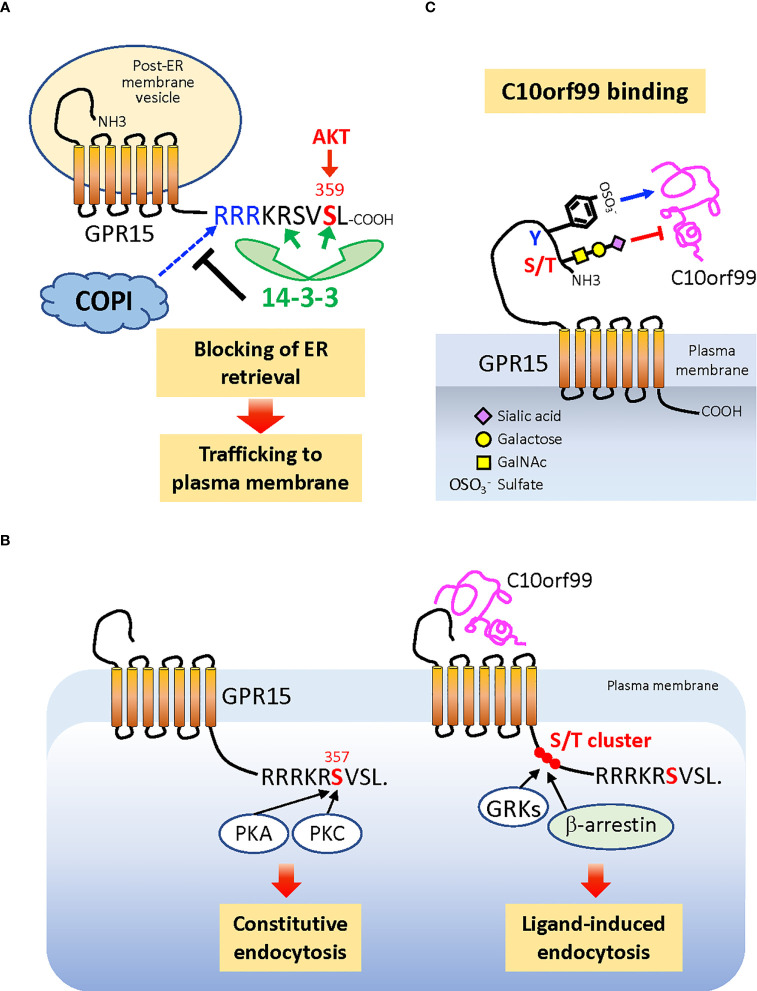
Posttranslational modifications that regulate cell surface expression and ligand binding of GPR15. **(A)** Phosphorylation of C-terminal penultimate Ser359 of the nascent GPR15 protein, which can be mediated by AKT, recruits dimeric 14-3-3 proteins to the C-terminus, which in turn blocks the binding of COPI to the adjacent di-Arg (RxR) motif. This prevents GPR15-loaded membrane vesicles from being retrieved back from the Golgi to the ER and allows GPR15 trafficking to the plasma membrane. **(B)** The cell surface level of GPR15 is also regulated by the constitutive (ligand-independent) endocytosis that requires phosphorylation of Ser357, which is the target of PKA and PKC. The more upstream Ser/Thr cluster that is commonly phosphorylated by GRKs and responsible for recruiting β-arrestin to many GPCRs is not required for the constitutive endocytosis of GPR15, while most likely required for the C10orf99-induced endocytosis. **(C)** The Tyr residues in the N-terminal extracellular region of GPR15 are sulfated ( OSO3 ^−^) while the Ser and/or Thr residues are O-glycosylated and capped with α2,3-sialic acids. Sulfated Tyr residues promote, while sialylated O-glycans inhibit, the binding of C10orf99 to the receptor. *ER*, endoplasmic reticulum; *PKA*, protein kinase A; *PKC*, protein kinase C; *AKT*, protein kinase B; *GRKs*, GPCR kinases; *COPI*, coatomer protein complex I.

Cell surface expression levels and ligand responsiveness of chemokine receptors are tightly regulated by various post-translational modifications (PTMs). The trafficking of *de novo* synthesized GPR15 to the plasma membrane is dependent on the phosphorylation of the penultimate Ser359 residue in the cytoplasmic C-terminal tail (120). Phosphorylation of Ser359, which can be mediated by AKT (121), induces binding of 14-3-3 proteins to the receptor (120). The 14-3-3 binding sterically masks the upstream di-Arg (RxR) motif composed of Arg352 and Arg354, which is the binding motif of COPI, a coatomer protein complex that mediates the retrieval of cargo protein-loaded membrane vesicles from the Golgi to the endoplasmic reticulum (ER) (122). Thus, Ser359 phosphorylation promotes the expression of GPR15 at the cell surface (**Figure 3A**).

In addition to receptor insertion into the plasma membrane, the cell surface expression of GPR15 is also controlled by endocytosis. GPR15 is constitutively internalized in the absence of exogenous ligand (C10orf99) stimulation (123). This constitutive endocytosis requires the phosphorylation of Ser357, which can be mediated by protein kinase A (PKA) or PKC, but does not require the upstream Ser/Thr cluster in the C-terminal tail, which is commonly phosphorylated by G protein-coupled receptor kinases (GRKs) and is critical for recruiting β-arrestin (124) (**Figure 3B**). Constitutively endocytosed receptors have been successfully utilized for the delivery of therapeutic agents to target cells; for example, drugs conjugated with specific antibodies to receptors (125, 126). Therefore, ligand-independent endocytosis of GPR15 provides a basis for developing interventions targeting this receptor in human inflammatory/immune diseases, where GPR15-expressing cells may play pathogenic roles (9–11, 47).

### Regulation of GPR15-C10orf99 interaction by Tyr sulfation and O-glycosylation

PTMs of GPR15 also regulate receptor-ligand interactions. Sulfation of Tyr residues in the extracellular N-terminus of chemokine receptors positively regulates the binding of chemokine ligands through electrostatic interactions (127, 128). GPR15 has also been recently found to be sulfated on Tyr residue(s), which enhances the binding of the endogenous ligand C10orf99 (21). It is interesting to note that latent membrane protein 1 (LMP1), which is encoded by EBV, induces tyrosine sulfation of CXCR4 through upregulation of tyrosylprotein sulfotransferase-1 (TPST-1) and promotes metastasis of cancer cells (129), a mechanism that could also be applied to GPR15.

GPR15-C10orf99 interaction is also regulated by O-linked glycosylation at the receptor N-terminus (21, 120). In contrast to Tyr sulfation, the O-glycans on the N-terminal Ser/Thr cluster negatively regulate ligand binding, which is at least in part due to the α2,3-linked sialic acid that caps O-glycans. This is similar to CCR7, in which sialylated O-glycans add steric hindrance to the receptor N-terminus to limit ligand binding (130). Consistent with their effects on ligand binding, Tyr sulfation and O-glycosylation of GPR15 differentially regulate the magnitude of receptor signaling (21). Thus, GPR15 represents a unique chemoattractant receptor in which two different PTMs on the N-terminus, Tyr sulfation and O-glycosylation, play contrasting roles in ligand binding and consequent signaling (**Figure 3C**), which is distinct from the reportedly cooperative roles of these two PTMs in the case of CCR5 (18) and CCR8 (20). The highly regulated glycosylation and sialylation during the differentiation and activation of T cells (131, 132) underpins the notion that the strength of the GPR15–GPR15L interaction is dynamically regulated by PTMs in both physiological and pathological conditions.

## Inhibitors of GPR15

There is currently no publication reporting small-molecule compounds that inhibit C10orf99-induced GPR15 signaling. Wang et al. (133) predicted the 3D structure of human GPR15 and applied structure-based virtual screening approaches to discover potential antagonists that could bind to the predicted active sites. By screening a chemical database consisting of 62,500 small molecules, they isolated a set of compounds that satisfied the threshold of a high docking score. However, their antagonistic effects on GPR15 signaling have not yet been demonstrated. Hayn et al. screened peptide libraries generated from human hemofiltrate, which essentially represents the entire blood peptidome, to identify novel endogenous ligands of GPR15 (134). Using primate lentiviruses, these authors discovered a C-terminal fragment of cystatin C (CysC95-146) that specifically inhibits GPR15-dependent entry of HIV-1, HIV-2, and SIV, but does not inhibit C10orf99-induced signaling. This indicates that CysC95-146 is an endogenous inhibitor of GPR15-mediated HIV and SIV infections that does not interfere with the physiological function of this GPCR. In another study by Guo et al. (135), *Gpr15* was found to be upregulated in CRC tissues, and silencing of *Gpr15* by siRNA inhibited the growth, migration, and invasion of CRC cells. These authors found that the expression of GPR15 is post-transcriptionally regulated by microRNA-1225 (miR-1225), the expression of which is significantly downregulated in CRC cells. Overexpression of miR-1225 caused suppression of GPR15 and inhibited the proliferation of CRC cells, suggesting its therapeutic potential (135).

## Conclusion and open questions

Over the past decade, a growing number of studies have indicated the involvement of GPR15 and its ligands in a variety of human immune disorders, making them promising therapeutic targets. In the past two decades, 45 drugs targeting chemokine receptors have been tested in clinical trials and only three have been approved by the Food and Drug Administration (FDA) (136). One of the major reasons for the poor success of chemokine receptor inhibitors may be the redundancy of chemokine-chemokine receptor interactions *in vivo*. In this regard, the unusual lack of cross-reactivity of GPR15 and C10orf99 with known chemokines and chemokine receptors, respectively, may offer an advantage for developing specific and clinically effective inhibitors. GPR15 is expressed in multiple cell types and plays pro-inflammatory or regulatory roles in both humans and mice. Further investigations to determine the relative contribution of specific cellular subsets in individual disease settings are necessary to enable the implementation of GPR15-targeted therapy. In this regard, development of conditional GPR15 KO mice likely facilitates elucidation of cell-intrinsic roles of GPR15. In addition, further discovery and characterization of new GPR15 ligands and regulatory mechanisms of GPR15 expression will expand our understanding of GPR15 biology in broader pathophysiology.

**The questions that remain open include**:

(1) GPR15 is strongly expressed by colon epithelial cells; however, the implication of epithelial GPR15 in disease is limited to HIV enteropathy and the growth of CRC cells. Studies in cell culture systems have demonstrated that chemokine/chemokine receptor signaling, such as CXCL8/CXCR1 and CXCL12/CXCR4, contributes to the maintenance of the epithelial barrier by stimulating the migratory repair process, termed restitution, of wounded epithelium (137, 138). It remains to be determined whether autocrine activation of GPR15 by C10orf99 in colon epithelial cells plays a role in barrier integrity through restitution. Also interesting will be whether GPR15 expression in colon epithelium is regulated by AhR stimulation by dietary and/or commensal metabolites.

(2) It is interesting that C10orf99 functions as an inducible “inflammatory” chemoattractant in psoriatic skin while this protein appears be a “homeostatic” chemoattractant in the colon where its expression was not altered by inflammation or the presence of commensal bacteria (7). It is still unclear what causes the increase in C10orf99 expression at the onset and/or during psoriasis. This is important since such regulatory mechanisms could also be applied to other C10orf99-expressing tissues including colonic and esophageal epithelia. Additionally, it is unknown whether the direct effects of C10orf99 on keratinocyte proliferation and inflammatory response are mediated by MRGPRs or other receptors.

(3) It remains open whether/how the different ligands such as C10orf99 and TME5 activate different signaling pathways (e.g., G-proteins) through GPR15. This may be cell context-dependent. It would also be interesting to investigate whether GPR15 undergoes different PTMs when expressed in different types of cells (e.g., lymphocytes, epithelial cells, and endothelial cells), which could potentially enable preferential interaction with particular ligands.

(4) Many studies indicate a correlation between smoking habit, hypomethylation of GPR15 DNA, and the increase of peripheral blood GPR15^+^ T cells; however, it is not clear how the smoking leads to the increase of GPR15 expression and whether the increase is specific to any T cell subsets (e.g., Tregs or Teffs). Those questions will be relevant for understanding the potential role of GPR15 in the smoking-related diseases.

## Author contributions

YO and SS wrote the manuscript and made figures. Both authors contributed to the article and approved the submitted version.
